# Supplementary figures and images for: Modelling and improving approach for carrying capacity in multi-modal super network by considering travel time
Source: PLoS One. 2025 Dec 22;20(12):e0339039. doi: 10.1371/journal.pone.0339039 (PMC12721534; doi:10.1371/journal.pone.0339039)

1:

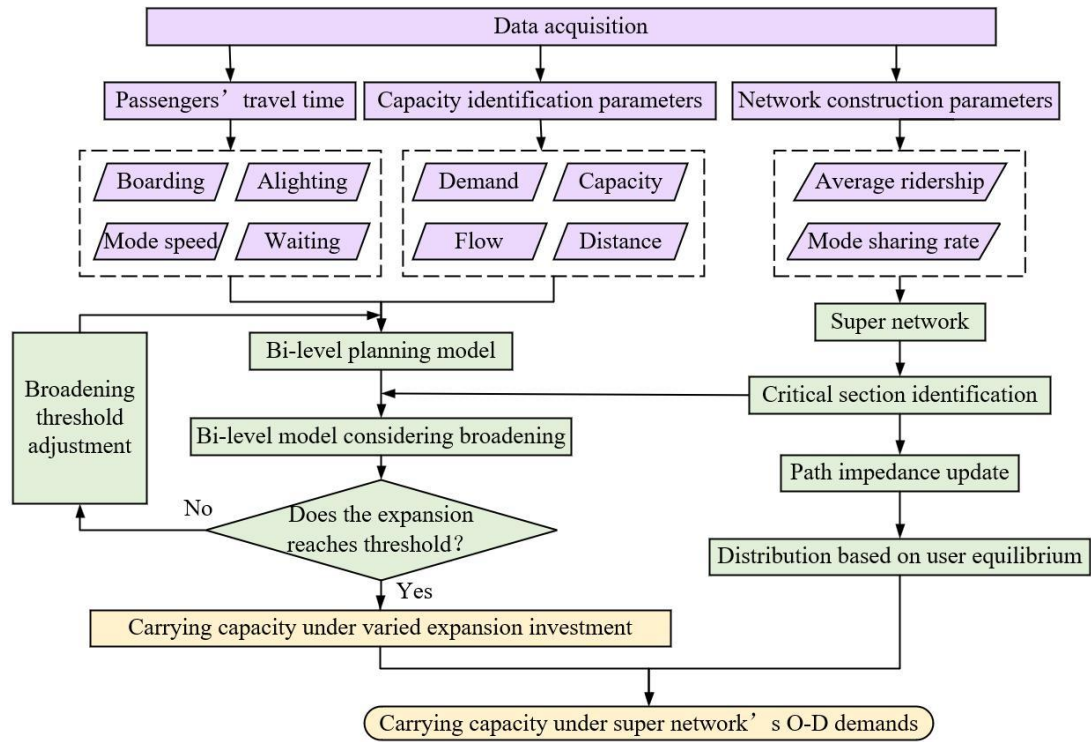

2:

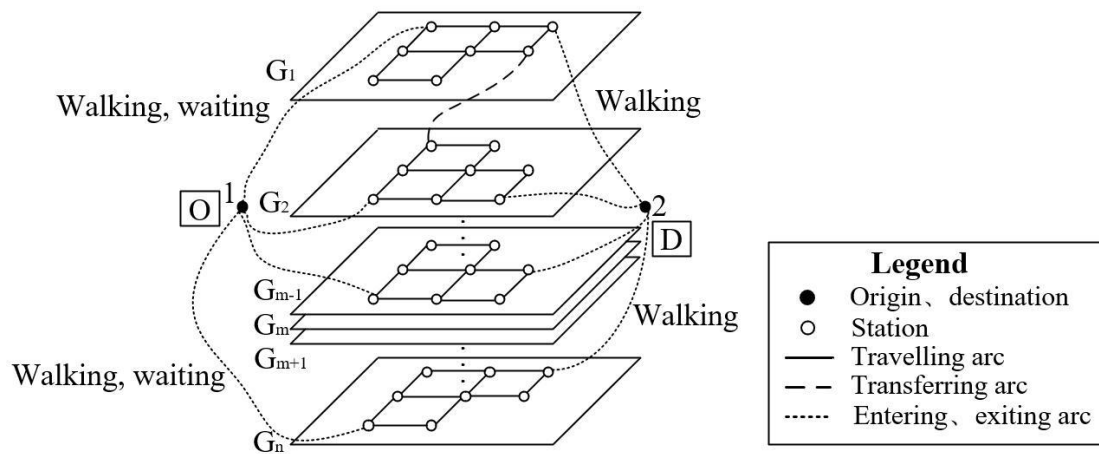

3:

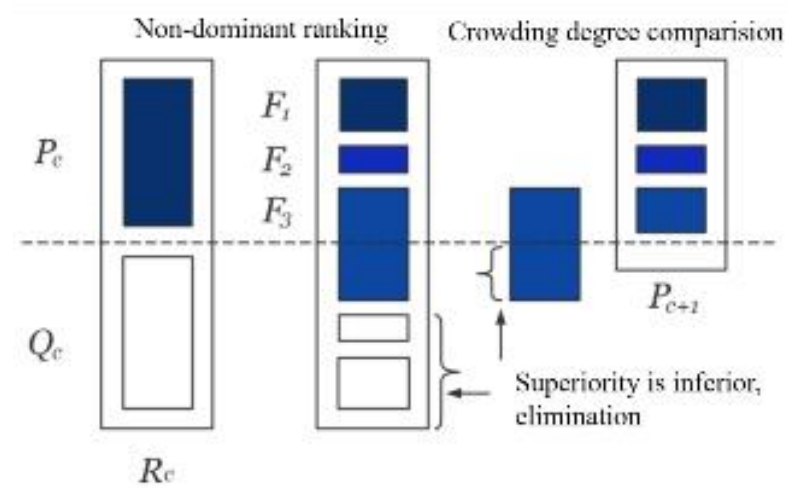

4:

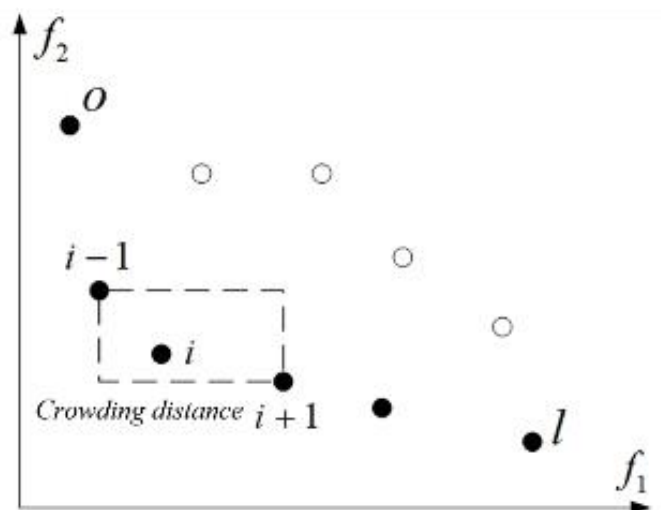

5:

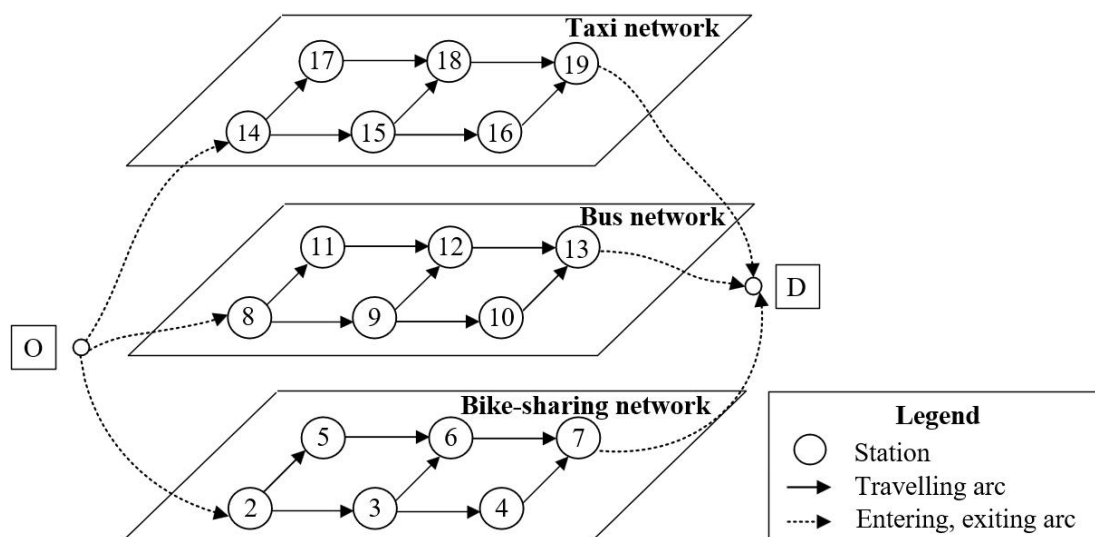

6:

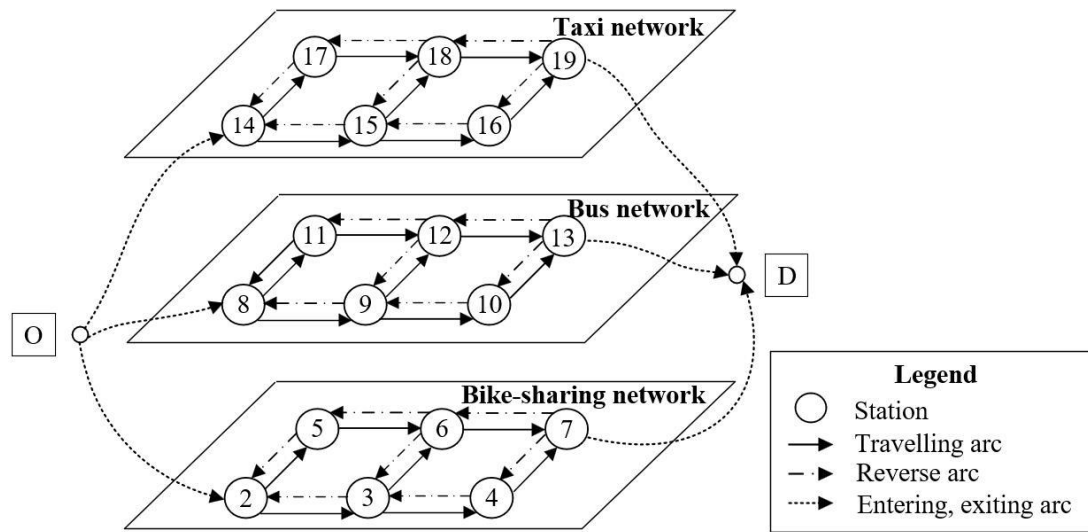

7:

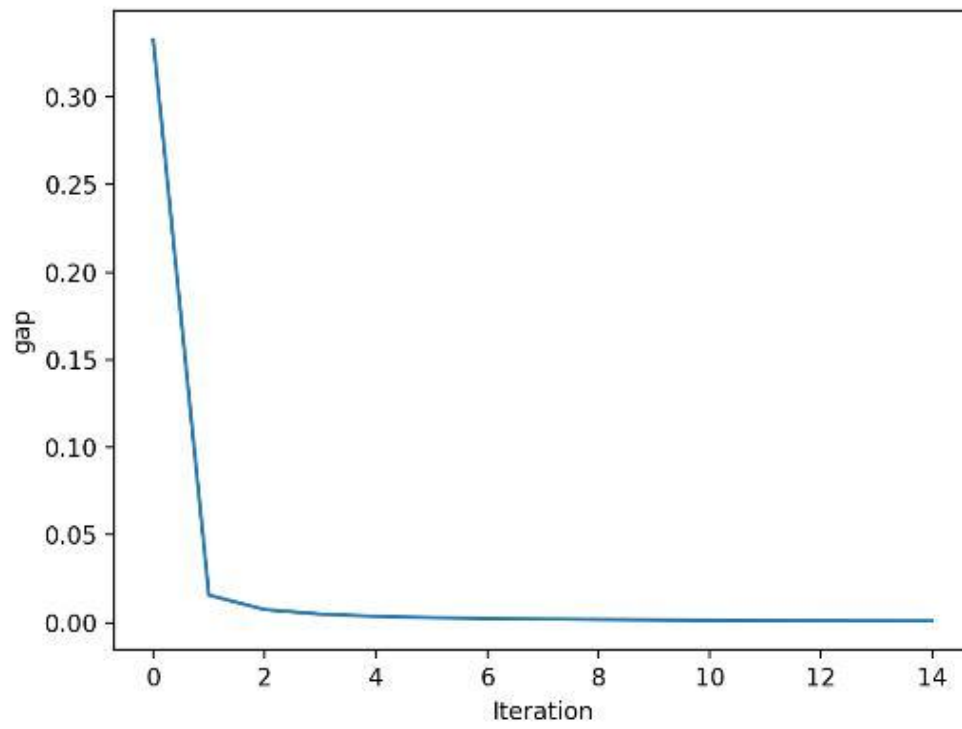

8:

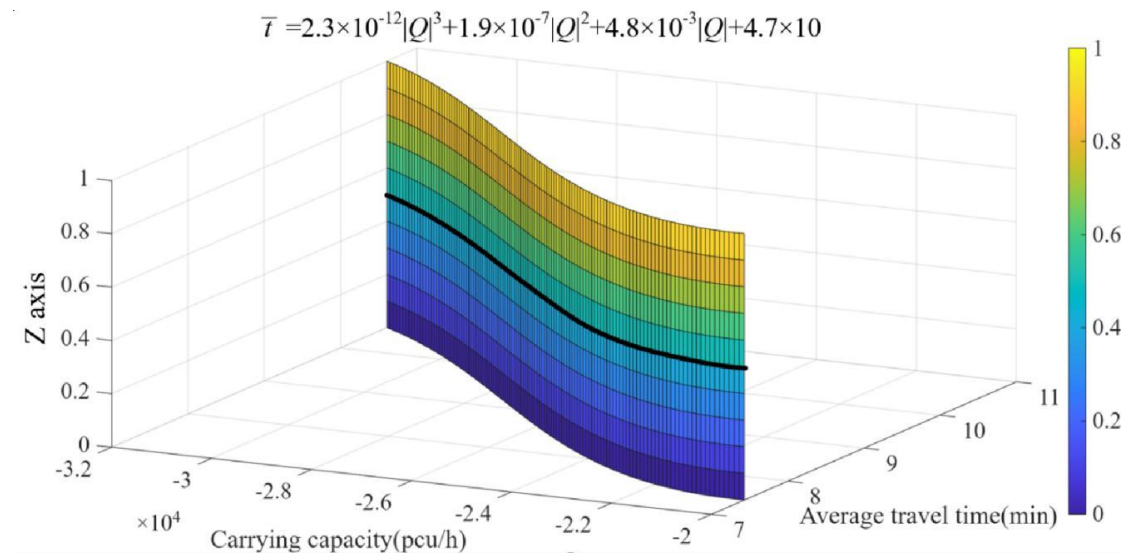

9:

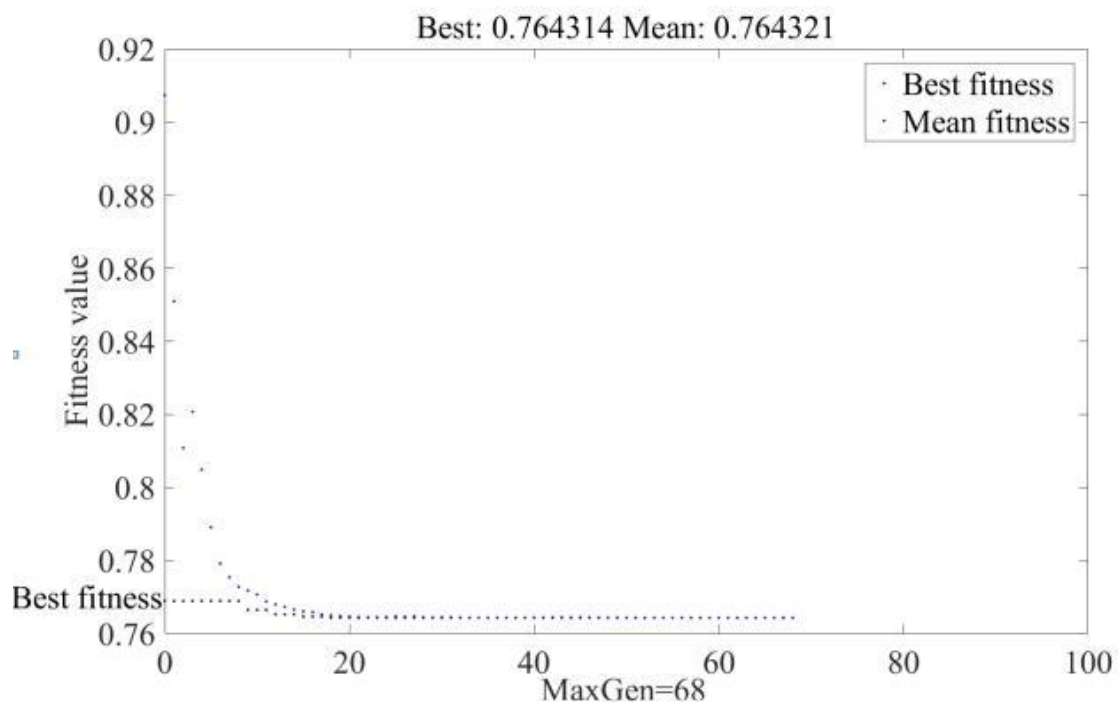

10:

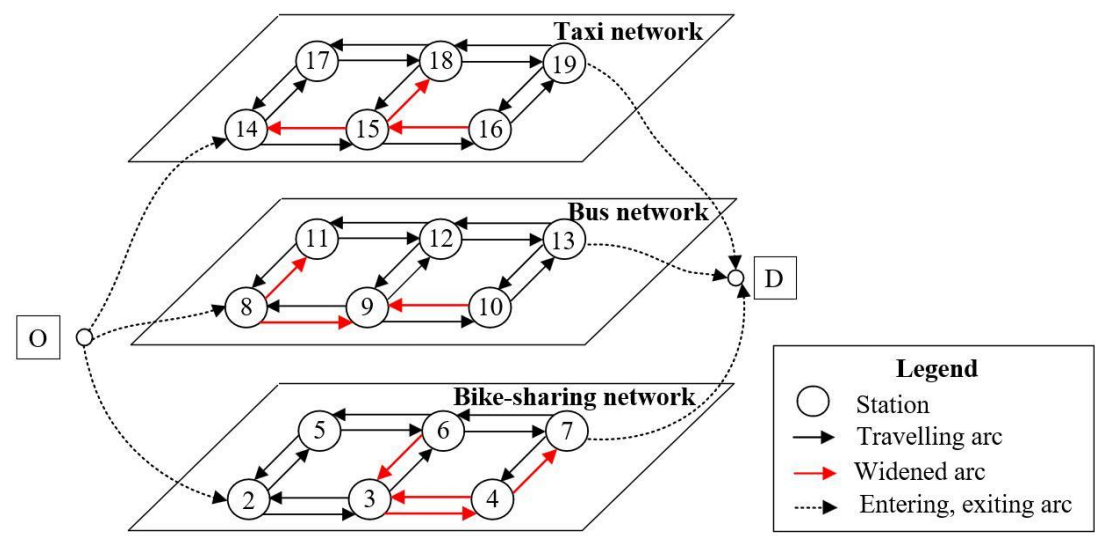

11:

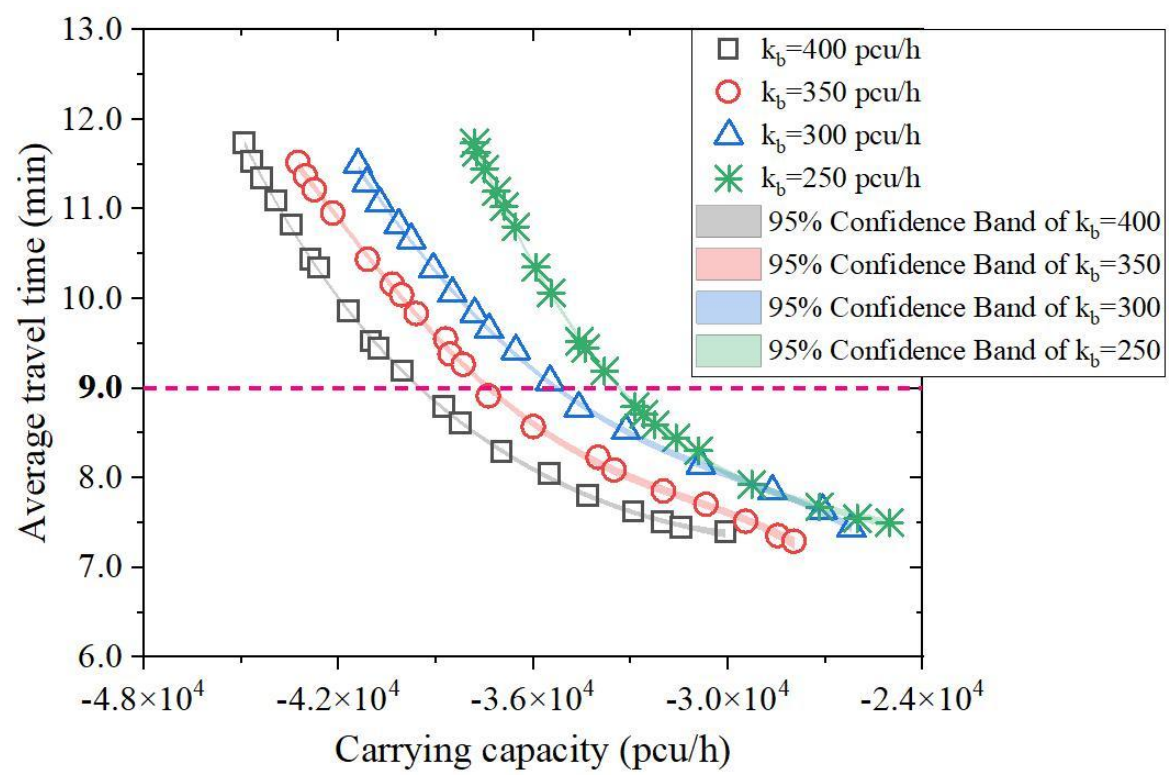

12:

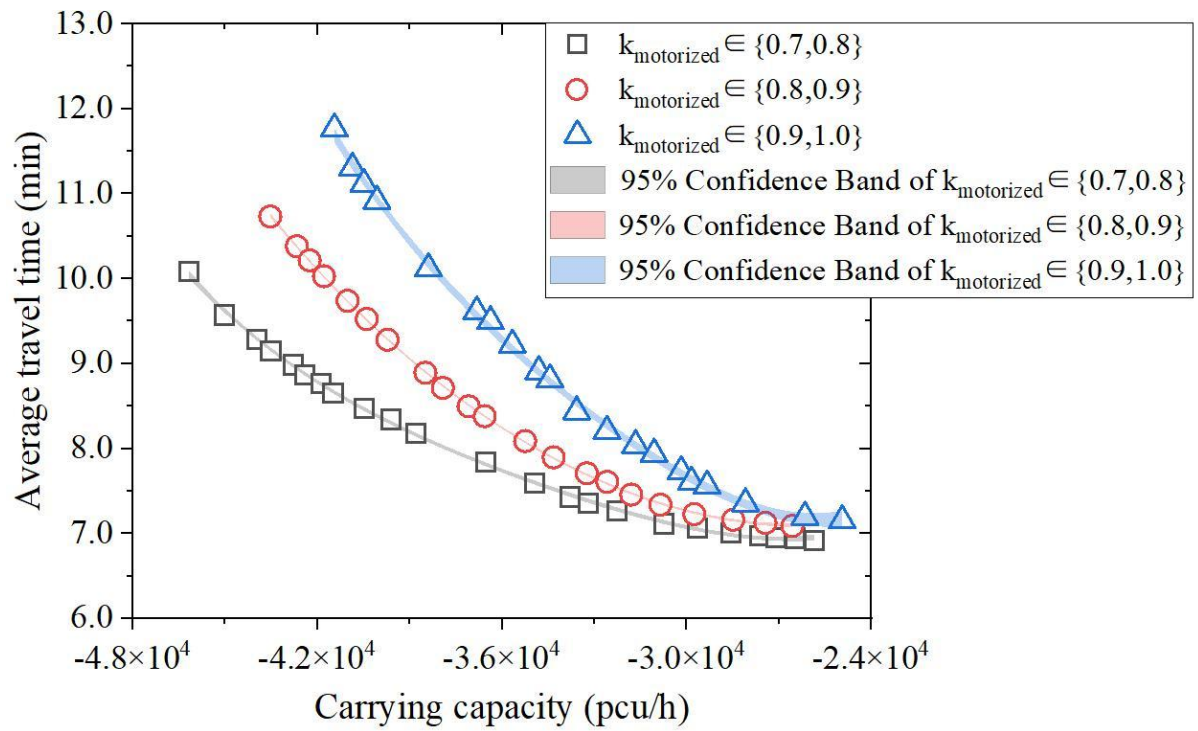

13:

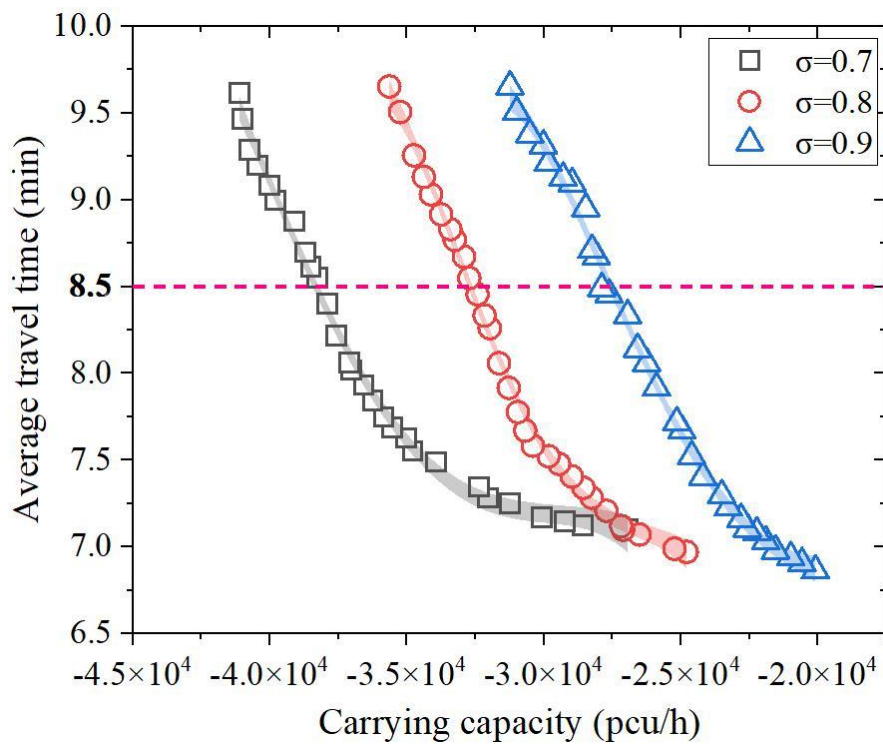

14:

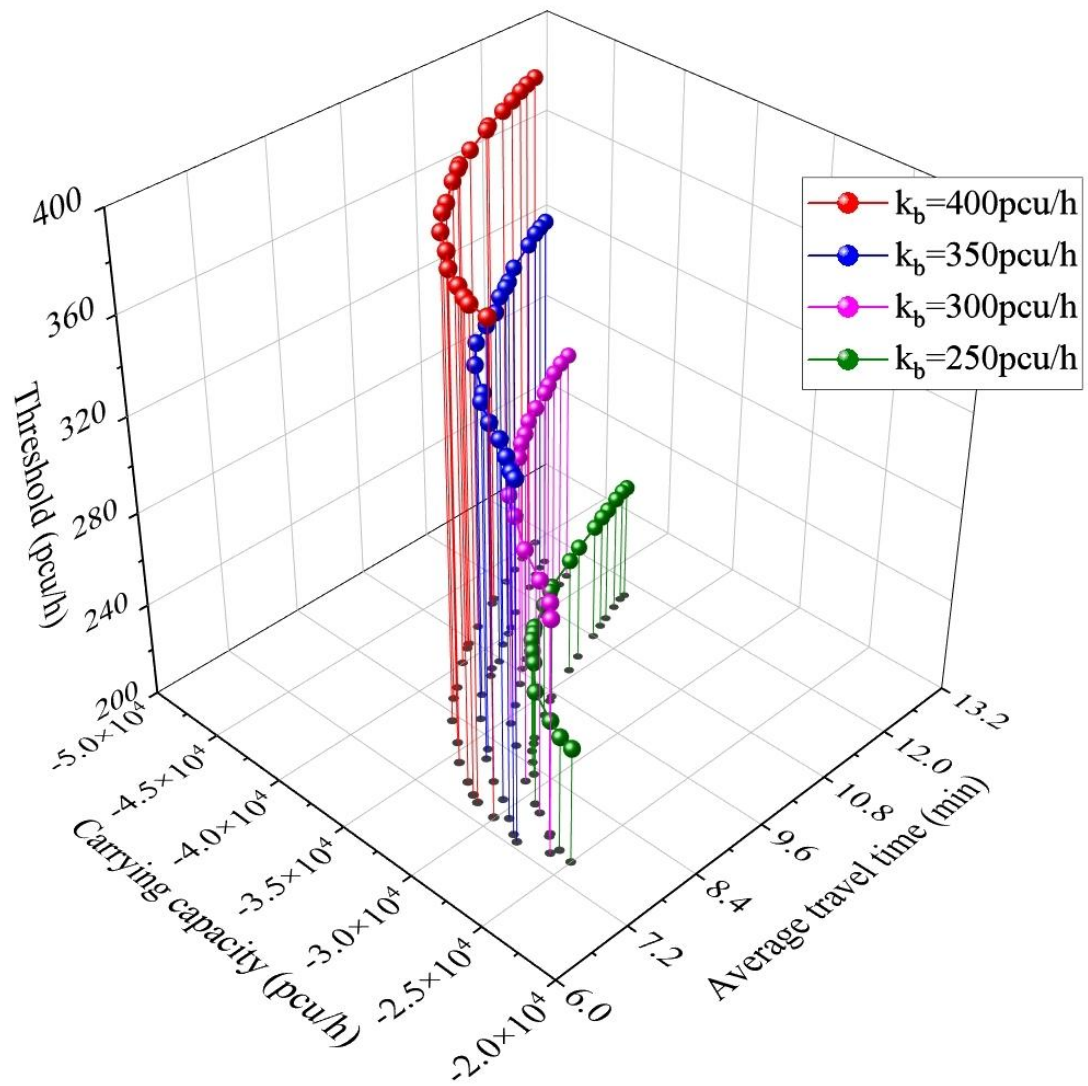

Supplement: S1 File — (PDF) [file pone.0339039.s001.pdf]
