# Supplementary material for: Modelling and improving approach for carrying capacity in multi-modal super network by considering travel time
Source: PLoS One. 2025 Dec 22;20(12):e0339039. doi: 10.1371/journal.pone.0339039 (PMC12721534; doi:10.1371/journal.pone.0339039)
Supplement: Attachment — Submitted filename: Response to Reviewers.docx [file pone.0339039.s004.docx]

**PLOS One**

**Ref. No.: PONE-D-25-28716**

**Title: Methodology of Improving Carrying Capacity of Multi-modal Super Network by Considering Travel Time**

Xiangyue Huang<https://orcid.org/0000-0002-9016-6105>^*^

College of Transportation Engineering, Chang'an University, Xi'an,

Shaanxi Province, China

Email: [hxyue019@163.com](mailto:hxyue019@163.com)

**REPLY to reviewer’s comments**

**To Reviewers:**

*Dear Reviewers:*

*This article has been moderate revised, I sincerely appreciate your kindness of the comments for the manuscript. 13 comments are all extremely precious suggestions for replenishing the explanation of the proper terms, pattern of results’ visualization, and standardizing writing skills in essay, besides, the details are also well challenged. After a buried, comprehensive reading process, I made up my mind to correct the mistakes which should have catered to the guidelines. Considering that both reviewers precisely pointed out the location of each problem in the essay, the revision version of the reply letter intends to provide the reference sources’ address directly, solutions are simultaneous provided in the revised* ***word*** *edition. In summary, the responds of both reviewers' comments which attached to the revision* ***word*** *edition are answered below. The revised essay adopted simple present tense, simple past tense and past perfect tense to narrate. The fonts of the first, second, and third-level headings in the article are Times New Roman's* 18*pt,* 14*pt, and* 11*pt respectively，figures and tables’ heading fonts are Times New Roman’s* 10*pt。*

*Sincerely,*

*Xiangyue Huang*

**To Reviewer #1**

**Reviewer #1:**

*This paper is well organized and provides a scaling scheme for multi-modal networks, which lays the foundation for alleviating congestion at the macro level. Nevertheless, this paper still has some shortcomings the following issues need to be further described:*
***1. There are virtually no color images throughout the text, and tables are uniformly presented with traditional light gray borders rather than the three-line tables.***
Response 1：

Thank you for your understanding and feedback. I appreciate your patience as I work to improve the visuals in my paper. The additional figures, especially the color picture in Figure 3, have really enhanced the overall presentation of my findings. Furthermore, updating the content of Figure 8 (original Figure 7), Figure 11 (original Figure 11), Figure 12, Figure 13 (original Figure 8) and Figure 14 (original Figure 12), not only clarified data but also brought consistency throughout sections of my research. I'm hopeful that these adjustments will meet or exceed expectations and contribute positively to our discussions moving forward. Please let me know if there are any other areas where you'd like further revisions or enhancements!

2. ***In “Data Collection” on pages 12 and 13, the font requirements for parameters and units are not uniform enough, and the font specification is somewhat arbitrary.***

Response 2：

Thank you very much for your wise comment, I carefully adjusted the units’ font to standardized font pattern (i.e., canceled the italic font) in “Notation” and “Data Collection” on pages 6 to 7 (original 5 to 6) and 12 to 13 after referring to the Font Column’s instruction in “Style and Format” of the exalted journal. Due to the fact that the variables in the text are not naturally changing (e.g., temperature), to distinguish variables or parameters compared with units, variables or parameters still retain the font of Times New Roman. The adjustments indicate the second column in Table 1 and Table 2, row 1 in Table 3-Table 7, column 2 in Table 3-Table 5, column 3,4 in Table 6, and column 3 in Table 7.

**The revised content is as follows in Red Rectangle (page 5-6, page 12-13):**

**-----------------------------------------------------------------------------------**

**（See Table 1 and Table 2 in “3.2 Notation”)**

**（See Table 3-6 in “5.2 Data collection”)**

**（See Table 7 in “5.3.1 Effective paths searched”)**

**-----------------------------------------------------------------------------------**

3. ***In the “Literature Review” on page 4, authors are advised to consider a separate narrative of the background of the study, including details and a summary of the established process of two-layer modeling.***

Response 3：

I appreciate your valuable feedback. In response to Reviewer 1’s suggestions, I have restructured the introduction of the bi-level model within subsection “2.3 Integration of Bi-Level Model and Carrying Capacity” in Section 2: Literature Review. This part has been divided into three subsections: "2.1 Traffic Assignment Solvers," "2.2 Carrying Capacity Models," and "2.3 Integration of Bi-Level Model and Carrying Capacity." Additionally, I've revised the research background included in section “2.3.” Thank you again for your insightful comments; they significantly improved this manuscript's quality.

**The revised content is as follows in Black-bold Font (page 2-3; page 4):**

**-----------------------------------------------------------------------------------**

**When a single vehicle is running on the road, due to the limitation of the surrounding driving environment, the traffic flow scale will be affected by the road capacity. When the network scale is wide, it may cause congestion on certain road sections[11, 12]. Based on the above fact, this essay carried on the reasonable lane combination scheme through the multi-modal network.** The contribution of this essay is mainly generalized in four aspects:

**(1)Considered the conservation of network traffic to establish a multi-modal network distribution model based on Method of Successive Algorithm, mainly to troubleshoot congested road sections and promote the balanced distribution of traffic.**

**(2)Established a bi-level planning model for integrated urban transportation modes, aiming to minimize travel time and maximize network carrying capacity, thus reducing traffic detours and improving driving efficiency.**

**(3)Obtained the flow distribution results by iterative method and incorporated them into the initial population of improved genetic algorithm, retained a method of screening out the best individuals for reproduction and keeping them to the next generation, designed a high-performance NSGA-II algorithm.**

**(4)Studied the relationship between road widening and cost consumption, added one of the goals to minimize investment cost, and proposed an improved multi-objective urban integrated transportation network optimization model.**

**This study provided the experience for the design of road improvement schemes for continuous-based network in the given case study and could serve as a good reference.**

**（See line 42-60 in “1. Introduction”)**

**Under majority of conditions, shared bikes riding are on the outermost side of roads, which lack the separation belt between non-motorized and motorized lanes, and could be apt to trigger friction among cyclists and drivers. Buses, due to their large size and lofty passenger capacity, ought to require sufficient clearance at stops for passengers to get on and off. To avoid potential risks, policymakers would rather take measures such as widening lanes to keep vehicles safe. The standard bi-level planning model is composed of the upper-level model section and the lower-level model section.**

**As illustrated in Fig 1., experts are still dedicated to improve network carrying capacity [27, 28]. The designed model regards the path impedance update as the lower-level model's target. Boarding and alighting time, average ridership, and mode selection probability in the mechanics diagram are also considered. In essay, lanes’ broadening scheme is set in the upper-level part. The upper-level part is regarded as a mathematical convex optimization problem containing two or three objectives with capacity constraints. If it is assumed that the road section could accommodate infinitely large traffic flow to allow all traffic demands to access, while ensuring traveling accessibility, an Augmented Lagrangian Function would be constructed to characterize the relationship between feasible traffic flow and capacity on sections, and an external penalty factor is incorporated into the extended objective function [29]. When the objective of travel time approaches the best solution, there must generate a corresponding feasible, optimized scheme which could be adopted. In the lower-level model, adopting the travel time of users between origin and destination as an impedance for network optimization could achieve the optimal state of network traffic distribution. However, when it comes to uncertainty factors such as road saturation and expansion cost, which make it hard to change the overall traveling status in the original models.**

**For the bi-level planning model covering multiple variables, it is difficult to obtain an exact solution by mathematical methods such as the Lagrange method and gradient descent. Therefore,** Zhang et al. [30] put forward a majorization approach of an advanced generic algorithm, **called NSGA-II (non-dominating sorting genetic algorithm), to solve the constrained multi-objective network design problem and express the optimal solution of the model with a Pareto frontier relationship.**

**（See line 103-126 in “2.3 Integration of bi-level model and carrying capacity”)**

**-----------------------------------------------------------------------------------**

4. ***In the Algorithm Design section on page 10, the key steps of the NSGA-II algorithm are not shown, i.e., the process of generating a new generation of populations from an elite strategy can be clearly illustrated with color pictures.***

Response 4：

I am profoundly apologetic for the oversight in the depiction of the critical process., I have taken your advise into consideration and then comprehensively depicted the specific steps of crowding distance ranking and elite strategy, the compensatory Figure 4 intuitively showed the role of elite retention strategy in transmitting and retaining good individuals in a new generation. The findings suggest that applying a strong retention strategy significantly influences not just the immediate outcomes but also shapes genetic diversity in future generations. By ensuring that high-performing individuals are retained, we can enhance overall population fitness and adaptability to changing environments. This approach offers promising avenues for further research into optimizing evolutionary algorithms and improving performance metrics across various applications.

**The revised content is as follows in Black-bold Font (page 11; page 12):**

**-----------------------------------------------------------------------------------**

**Step 3: Crowding distance ranking: After completing the non-dominated sorting, we select the top-performing individuals from F_1_ and F_2_ to form the next-generation Parent population P_(c+1)_. To maintain numerical parity with the original Parent population P_c_, it becomes evident that all F_3_ members cannot be accommodated within P_(c+1)_. Subsequently, we perform a crowded distance ranking on the Child generation of F_3_. This process aims to eliminate local maximum and minimum values.**

**Fig.4 The algorithm of elite strategy**

Step 4: The implementation of elite strategy (Fig 3) and the determination of crowding degree; **the purpose of the“elitist preservation strategy”is to prevent the loss of the optimal individual of the current group in the next generation, which would lead to the genetic algorithm not converging to the global optimal solution. The core scheme is to directly copy the excellent individuals of the current generation into the next generation without pairing and crossing. In other words, the optimal individuals of the previous generation have taken the outstanding genes, which are worth preserving.**

**（See page 11, line 252-264 in “4.2 The Non-dominated Sorting Genetic Algorithm II”)**

**Step 5: Crowding degree’s estimation; the resulting set indicates the first level non-dominant layer of the population, which ignores the identified individual; repeating this step to the second level non-dominant layer until the entire population is stratified as shown in Fig 4.**

**（See page 12, line 267-269 in “4.2 The Non-dominated Sorting Genetic Algorithm II”)**

**-----------------------------------------------------------------------------------**

5. ***The authors state that "when the road saturation increases from 0.6 to 0.9, cyber carrying capacity increased by 13.97%, and average travel time of passengers reduced by 66.02%." What is the relationship between road saturation and road carrying capacity, and how does it significantly reduce passenger travel time? This is very confusing, please clarify.***

Response 5：

I sincerely apologize for the inaccuracies in my writing., by reexamining the conclusion, it must be found out that the mathematical connection between average saturation (level of service, LOS) and travel impedance are re-clarified by Figure 13 and Table 9. That is - “when the road saturation increases from 0.7 to 0.9, the plural modulus of carrying capacity declines by 27.1%” is true. The influence on the other objective, passenger travel time, is reflected in the change of the positive difference in the initial solution set according to the output's initial order of best solutions. As adjustments are made to optimize system performance, improvements can be observed not only in overall efficiency but also in individual travel experiences for passengers. By prioritizing certain routes or methodologies based on real-time data and feedback loops from previous outcomes, we can create a more reliable transportation model that minimizes delays while ensuring comfort and accessibility for all users involved. This iterative process exemplifies how effective strategy refinement leads directly to enhanced service levels mechanistically aligned with user needs.

**The revised content is as follows:(page 19)**

**-----------------------------------------------------------------------------------**

## 5.5.1 Downgrade the level of service. As described earlier, no matter what the traffic state was like, there would always be a corresponding optimal solution. Moreover, the scattered results would move further away from the origin as the saturation promoted by 0.1, which means that the carrying capacity would present a downward trend. **Through taking a portion of Pareto Frontier, the fitting curve of Pareto fronts and their 95% confidence interval belt under three LOS conditions could be clearly presented. As shown in Fig 13.**

**Fig 13. Pareto fronts of stable LOS flow states**

**Results show that the downgrading of average saturation elevates the carrying capacity of the network to a certain extent. For example, when the dependent variable along y axis equals 8.5min, the corresponding carrying capacity modulus indicates 3813.7pcu/h, 3278.4pcu/h, 2779.6pcu/h respectively from left to right. and the annotated diagram presents the overall result (with dependent variable outweighing 7.0): |*Q*_σ=0.7_| > |*Q*_σ=0.8_|> |*Q*_σ=0.9_|. In order to let correlation coefficient modulus R^2^ according to the optimal solution close to 1, while combining the inherent curvature of Pareto Frontier. After error weaken in Origin Apps' Simple Fit, we get a series of quartic polynomial functions, label them as scientific notation with one decimal place retained, then fill in Tab 9.**

**Table 9. Objective function’s average Pareto front of different LOS.**

| **σ** | **Pareto fronts** | **Satisfied fronts** | **Changing trend of Polynomial fitting function** |
| --- | --- | --- | --- |
| 0.7 | 28 | 28 | y=-1.4×10^-16^x^4^‒2.0×10^-11^x^3^‒ 1.0×10^-6^x^2^‒2.3×10^-2^x+1.8×10^2^ |
| 0.8 | 29 | 28 | y=-5.5×10^-16^x^4^‒6.6×10^-11^x^3^‒2.9×10^-6^x^2^‒5.6×10^-2^x‒4.0×10^2^ |
| 0.9 | 30 | 30 | y=-2.4×10^-16^x^4^‒2.3×10^-11^x^3^‒7.7×10^-7^x^2^‒1.1×10^-2^x‒5.5×10^-1^ |

**（See line 390-405 in “5.5.1 Downgrade the level of service”)**

**-----------------------------------------------------------------------------------**

6. ***In the author's abstract, 9 road segments are mentioned as research objects, but 10 road segments are mentioned in the conclusion, please explain.The author carefully checked and rewrote the conclusion section.***

Response 6：

Glad to receive any skepticism of contradictory rhetoric about my essay, “9 road segments” distribution results in “5.3.1 Effective paths searched” are correct, and through careful recheck and verification, some errors in the expression of results in the Abstract and Conclusion are picked out and corrected. The previously mentioned “10 road segments” are transformations that are considered in the expansion scheme, not the flow distribution results. These transformations aim to optimize traffic management and improve overall vehicle flow efficiency by analyzing key metrics such as speed, density, and congestion levels across different sections of the roadway network. By focusing on these specific segments during planning stages, city planners can implement targeted strategies to mitigate bottlenecks and enhance connectivity between crucial areas within urban settings.

**The revised content is as follows (page 2; page 21):**

**-----------------------------------------------------------------------------------**

**This experiment attains 9 effective hyper-paths with traffic flow and travel impedance attributes attached to each hyper-path. The scatter chart results show that the plural modulus of carrying capacity decreases with travel time’s descends; when the road saturation increases from 0.7 to 0.9, the plural modulus of carrying capacity declines by 27.1%. Meanwhile, raising the upper limit of expansion would slightly lift the carrying capacity in the proportion of 15.7%.** This research could provide a reference for the majorization of multi-modal cyber flow distribution and also has indirect significance for route planning to a certain extent.

**（See page 2, line 15-21 in “Abstract”)**

Utilizing the non-dominated sorting genetic algorithm II to acquire the pareto fronts under different widening conditions, for this purpose, the flow distribution results achieved the optimal targeted weight coefficient by algorithm iteration. **On the basis of that, the multi-objective and bi-level planning model was figured out through NSGA-II algorithm. Case study proved that traffic demand, average travel time, and whether the impedance function parameters change were all major factors affecting the carrying capacity’s value. The plural modulus of carrying capacity in the super network grew accompanied with average travel time’s rose. The sensitivity test varified its high-consistency transformation capability in comparison to the benchmark experiment.**

**However, there are still deficiencies in this essay, on account of quantitative factors are restricted by the accumulated process, such as economic development level and resident population, are difficult to be solely expressed by linear equations. Meanwhile, further study should explore and validate more parameters’ definition in the transfer arcs of a directed network. In addition, index optimization and model accuracy are the key points to be improved in the future.**

**（See page 21, line 415-426 in “6. Conclusion”)**

**-----------------------------------------------------------------------------------**

7. ***The author has mentioned the traffic carrying capacity many times in this paper, its unit is a negative number, and its order of magnitude and value are also various, so it is suggested to unify. Figure 12 What is the significance of the suggested use of a line chart?***

Response 7：

Thank you for your doubt, the negative value of the carrying capacity could be expressed scientifically for unification. The value then adopts scientific notation method of 10 to the third, real number part is retained to one decimal place in the revised edition, and its unit is pcu/h.

Besides, The significance of using line chart could be divided into three counterparts: firstly, to display the upward, downward or stable trend of the Pareto fronts; then, it assists to distinguish the differences of Pareto fronts under every 50-unit varied threshold boundary of widening; at last, line chart is able to make contributions to predict the tendency style of the line connected pattern by the Pareto scatter diagram of the two objective functions. In view of the lack of description of the line graph in the seventh problem, this paper explains the purpose and significance of using the line graph to describe the iterative process on the basis of the original text. In programming algorithms, the generation of line charts could visually present the optimization process and convergence tendency of the algorithm in each iteration, especially in the process of non-dominant solution set hierarchy, there exists a step of updating the dominance degree of individuals, thereby waiting for the algorithmic machine to select all levels of Pareto frontier, and laying the foundation for a new population’s production in the next step.

The diagrams which contain the negative values are in Figure 8(original Figure 7), Figure 11(original Figure 11), Figure 12, Figure 13(original Figure 8), Figure 14(original Figure 12).

**The revised content is as follows (page 16; page 19; page 20-21):**

**-----------------------------------------------------------------------------------**

**As depicted in Fig 7, the visualization of the iteration process is a scientific way to verify the effectiveness of the distribution algorithm: the relative gap of the objective output gradually approaches 0. Through the average of a series of auxiliary points in the iteration, the final iteration step is obtained after solving the auxiliary planning problem for a certain period of time, and the output is constantly corrected until it approaches the correct result.** The iteration of traffic volume algorithm is to narrow the gap as well as quicken the calculation. The final iteration curve stabilizes at 5×10^-2^ of gap value, and the total number of iterations has reached so far. **In programming algorithms, the generation of line charts could visually present the optimization process and convergence tendency of the algorithm in each iteration, especially in the process of non-dominant solution set hierarchy, there exists a step of updating the dominance degree of individuals, thereby waiting for the algorithmic machine to select all levels of Pareto frontier, and laying the foundation for a new population’s production in the next step.**

## **（See line 313-323 in “5.3.1 Effective paths searched”)**

**-----------------------------------------------------------------------------------**

**To Reviewer #2**

**Reviewer #2:**
***1. Lack of Clarity in Abstract, (e.g., "free-floating roads" on Page 1) and illogical "zero travel time" contradict real traffic conditions. Define key terms (e.g., "uncongested roads") and revise contradictions (e.g., "low-delay scenarios").***
Response 1：

In appreciation of your guidance on abstract composition, I have diligently implemented your suggestion to redefine the terminological constructs within the research narrative, specifically altering"congested road" and"non-congested road" to enhance clarity. Moreover, I have meticulously removed the contradictory expression "low delay scenarios" to ensure a cohesive and unambiguous presentation of the study's findings. “Free-flow travel time” as the total travel time’s coefficient terms which are mentioned in essay are gathered in the surveyed data.

**The revised content is as follows (page 2):**

**-----------------------------------------------------------------------------------**

**For the sake of achieving the mensuration of network carrying capacity under regular even congested road conditions with crowded vehicles, passengers or cyclists in a median-scale network, this study examined the ideal travel time of a passenger or cyclist in the hybrid-congested roads (congested and non-congested road accounts for half), being the initial accumulated values for the independent variable in Aggregated Functional Equation. Besides, the various road section’s capacity was examined, together with the total travel time accumulated accordingly, and the capacity limitation took effect to alleviate the heavy load of vehicles on partial road sections.** This study proposed a multi-objective bi-level planning model on the basis of the original capacity constraint model to address the potential congested problem, the model optimized three aspects in the schematic diagram: carrying capacity, average travel time, and expansion cost.

**（See line 7-15 in “Abstract”)**

**-----------------------------------------------------------------------------------**

***2. Assumption 3 (Page 11) ignores transfer time between modes, a critical factor in multimodal networks. Incorporate transfer time or justify its exclusion with references.***
Response 2：

The revised version choose to ignore the critical factor of transfer time in multi-modal networks - and the basis for its exclusion is explained with literature references in Assumption 2 on page 6. Firstly, for complicated combined trips, transfers between modes spend passengers’ or cyclists’ physical efforts; secondly, during the transfer process at stations, the varying durations of multiple modes of transportation pose greater challenges to the accumulation of total path costs. Therefore, we temporarily disregard additional travel route options generated by transfers. In literature records, most scholars choose to weaken the concept of "transfer cost", or take transfer time as a random disturbance term into the path cost. In reference[31], “the transfer walking time is included in the bus path impedance, but the transfer behavior is not explicitly modeled”; in reference[32], “In DTA, the time among transfers is absorbed by the dynamic impedance function”.

**The revised content is as follows (page 6; page 24):**

**-----------------------------------------------------------------------------------**

**(2) This research would not absorb certain physical parameters in the paper, such as transit time, driving speed, and vehicle weights; only the waiting and walking time on the station connection arcs, traveling arcs are included, indicating that any transferring time equals zero [31, 32];**

**（See line 139-141 in “3.1 Research scenario”)**

**Added references:**

**[31] Lam W, Morrall J. BUS PASSENGER WALKING DISTANCES AND WAITING TIMES: A SUMMER-WINTER COMPARISON[J]. Transportation Quarterly, 1982, 36(3):407-421.** DOI:10.1007/BF00167931

**[32] Peeta, Srinivas, and Athanasios K. Ziliaskopoulos. Foundations of dynamic traffic assignment: The past, the present and the future. Networks and spatial economics. 2001: 233-265.** DOI:[10.1023/A:1012827724856](http://dx.doi.org/10.1023/A:1012827724856)

**（See line 541-545 in “References”)**

**-----------------------------------------------------------------------------------**

***3. Data and Visualization Issues, Inconsistent units in Tables 3-6 (e.g., time in hours but values too small; 0.13h ≈ 7.8 min). Figure 7 (Page 20) fails to clearly illustrate correlations, standardize units (e.g., minutes) and enhance graphs with labels/trend analysis.***
Response 3：

Thanks extremely for your useful suggestion, I have revised the unit of average travel time in the table 3-6 from “h” to “minute” considering drivers’ capability based on units’ conversion law, so as to facilitate the comparison of the variation distinction of solution set under two objectives. The adjustments indicate the second column in Table 1 and Table 2, row 1 in Table 3-Table 7, column 2 in Table 3-Table 5, column 3,4 in Table 6, and column 3 in Table 7. Meanwhile, after examining the number of reserved places after the decimal point, I decided to yield to the unified regulation in the literature together with your advice in my paper. In addition, the black trend line and standardize units are labeled to enhance graph in Figure 8 (original Figure 7).

**The revised content is as follows (page 13-16, line 286-322):**

**-----------------------------------------------------------------------------------**

The management system has greatly improved the efficiency of transport by gathering cyber-infrastructure issues. **Each mode controlls the same road combination topology**, any one of which owns fixed attributes that belongs to every road section, such as the users' average travel time, free-flow travel time, **and road section's distance attributes, partly of them are irrelevant variables to model calculations.**

**Taking the two-way point-to-point of home-work and home-school travel as the basis, the cost of a single car commute spends at least 15 minutes. Based on that, many scholars[33, 34] have put forward the concept of time value in order to guarantee the normal operation of the city.** Detailed information on the journey is given in Table 3 to Table 6.

## **（See page 13, line 286-293 in “****5.2 Data collection”)**

## **(See Table 1 and Table 2 in “3.2 Notation”)**

## **(See Table 3-6 in “5.2 Data collection”)**

## **(See Table 7 in “5.3.1 Effective paths searched”)**

## 5.3.2 Pareto fronts' distribution. **Fig 8 illustrates that some variables, such as *v_m_, k_m_*, *A_m,l_, B_m,l_,* et al, which are deemed as irrelevant variables, they have nothing to do with the final results’ distribution. The theoretical Pareto fronts, which contain both carrying capacity and individual travel time, are ought to form a smooth curve surface in three-dimensional space (Target 1, 2 are deemed to x and y axes respectively). As depicted in the figure below, the black-color curve represents the fitting curve of the optimal scattered point solution set, the fitting function is as follows (one decimal place retained after radix point): y=2.3×10^-12^x^3^+1.9×10^-7^x^2^+4.8×10^-3^x+4.7×10; and the correlation coefficient of the objective solution is 9.8×10^-1^. In summary, as individual users’ travelling endurance decline, the plural modulus of the first objective is also dropping.**

**Fig 8. Curved surface of multi-objective functions' optimization**

**(See line 326-335 in “5.3.2 Pareto fronts' distribution”)**

When the number of iterations reaches 68, the calculation results tend to be stable. **The best fitness is between 7.6×10^-1^ and 7.8×10^-1^.** Attain the matlab data for the genetic algorithm, and combine it with the expression of the bi-objective function. **Weight coefficient calculation results show that when *ω*_1_ approaches 0.2 and there is a slight surplus of 0.8 for weight coefficient *ω*_2_, the optimal value of the compromise solution could be attained.**

**(See line 341-345 in “5.3.3 Linear fitting”)**

**Added references:**

**33. Fan Yuhang. Research on the risk attitude of travel time and its impact on the value of time reliability[D]. Kunming University of Science and Technology, 2021.** DOI:10.27200/d.cnki.gkmlu.2021.001041.

**34. Zhao Weitao. Research on model of time value of commuter travel[D]. Beijing Jiaotong University, 2013.**

**(See line 547-550 in “References”)**

**-----------------------------------------------------------------------------------**

***4. Add parameter sensitivity tests or cite benchmark studies; include comparative experiments.***
Response 4：

You're welcome! I'm glad to hear that you found my comments useful. In the context of parameters sensitivity tests, it's crucial to delve deeper into how each parameter influences outcomes. For instance, understanding the upper limit for expansion can help us gauge possible future demands on infrastructure. Meanwhile, assessing the proportion of motor vehicles allows for a better prediction of traffic patterns and potential congestion points within the network capacity framework. This comprehensive analysis will ultimately aid in optimizing our transportation systems effectively. They contribute to the network carrying capacity’s results.

**The revised content is as follows:(page 17; page 18; page 19)**

**-----------------------------------------------------------------------------------**

**- As shown in Fig 10. Apart from non-expandable black-marked sections , the rest red-marked 10 sections are all availably flexible to expand.** The scheme includes station 15 to station 14; station 15 to station 18; station 16 to station 15; and station 8 to station 9,11; station 10 to station 9. In addition, the abstracted bike-sharing network covers 4 expandable road sections: station 4 to station 3,7; station 6 to station 3; station 3 to station 4.

**（See line 347-350 in “5. 4 Sensitivity analysis”)**

## - **It is worth mentioning that the calculation results of the improvement scheme in this paper are based on the above section-updated super network. The specific measure is to expand the capacity of the road mentioned above. Changes in the target results of this scheme before and after widening are partly shown in Table 8 [35], the essay adopted 25% value of the benchmark experimental threshold as an increment to launch the sensitivity analysis of the model. From these figures, there is a positive increment between bi-level planning model and the benchmark experiment**.

**（See line 353-358 in “5. 4 Sensitivity analysis”)**

**- 5.4.1 Upper threshold. As shown in Fig 11, the promotion of widening threshold every 50 units mildly elevates the carrying capacity’s modulus within the dependent variable ranging from 7.0 to 12.0. The fitting curve of Pareto fronts and their 95% confidence interval belt of the quartic polynomial function. For example, when the dependent variable along y axis equals 9.0, the corresponding carrying capacity’s modulus indicates 3967.2pcu/h, 3761.3pcu/h, 3580.5pcu/h and 3345.8pcu/h separately from left to right, and the annotated figure presents the overall result (with dependent variable outweighing 7.0): |*Q*_kb = 400_| > |*Q*_kb = 350_| > |*Q*_kb = 300_| > |*Q*_kb = 250_|.**

**Fig 11. Pareto frontier for every 50 units change in the upper threshold**

**（See line 368-376 in “5.4.1 Upper threshold”)**

**- 5.4.2 Mobility rate. With the increasing occupation of non-motor vehicle lanes by motor vehicle lanes on roads, aiming at simulating and distributing vehicles in the super network, this paper further compares while under three different levels of mobility rate: 0.7-0.8, 0.8-0.9 and 0.9-1.0, the contribution value of multi-objective function to carrying capacity. In Fig 12, k_motorized_ is equal to the sum of sharing rates of cars and buses - k_taxi_ (k_1_) plus k_bus_(k_3_). Obviously, the contribution value of objective function to carrying capacity in three situations shows the same fluctuated law, which is mainly due to the fact that the number of motor vehicles is only related to the network topology and traffic model split, thus resulting in the fixed effects affecting the prediction way. In a word, for different motor vehicle ratios, the modulus of carrying capacity contribution value gradually decreases with the reduction of travel time. The three fitting curves ultimately stabilizes at 2.6×10^4^pcu/h, 2.7×10^4^pcu/h and 2.5×10^4^pcu/h respectively.**

**Fig 12. Pareto frontier for every 0.1 units change in the motorized ratio**

**（See line 377-388 in “5.4.2 Mobility rate”)**

**-----------------------------------------------------------------------------------**

***5. Abstract claims "13.97% capacity increase when σ=0.6→0.9", but the conclusion (Page 25) states "|Q| at σ=0.9 is lower than at σ=0.7/0.8". verify data consistency and revise conclusions accordingly.***
Response 5：

Extremely grateful for your meticulous suggestion, the calculation results in “Abstract” is true, and the calculation results in “6. Conclusion” is false. I first obtained the average optimal solutions at saturation levels of 0.7, 0.8, and 0.9. Subsequently, I summarized the outcomes of two key objective functions: Objective Function One (carrying capacity) and Objective Function Two (travel time). By comparing these outcomes, we identified the increasing rates of carrying capacity (27.1%) and travel time (0.7%) under the service level of 0.7 (non-congested condition) condition compared with the average saturation equals 0.9 (congested condition). These consistent findings were incorporated into the revised “Abstract” section and were summarized in “6. Conclusion” section. All results are retained by one decimal place.

**The revised content is as follows:(page 2; page 21; page 20)**

**-----------------------------------------------------------------------------------**

- The scatter chart results show that the **plural modulus** of carrying capacity **decreases** with travel time’s **descends**; when the road saturation increases from **0.7** to 0.9, the **plural modulus of** carrying capacity **declines** by 27.1%.

**（See line 16-18 in “Abstract”)**

- **Case study proved** that traffic demand, **average** travel time, and whether the impedance function parameters change **were all major factors** affecting the carrying capacity’s **value**. The **plural modulus of carrying capacity** **in** the super network **grew accompanied** with average **travel time’s rose. The sensitivity test verified its high-consistency transformation capability in comparison to the benchmark experiment.**

**（See line 418-421 in “6. Conclusion”)**

**Fig 13. Pareto fronts of stable LOS flow states**

- **Results show that the downgrading of average saturation elevates the carrying capacity of the network to a certain extent. For example, when the dependent variable along y axis equals 8.5min, the corresponding carrying capacity modulus indicates 3813.7pcu/h, 3278.4pcu/h, 2779.6pcu/h respectively from left to right. and the annotated diagram presents the overall result (with dependent variable outweighing 7.0): |*Q*_σ=0.7_| > |*Q*_σ=0.8_|> |*Q*_σ=0.9_|.**

**（See line 395-400 in “5.5.1 Downgrade the level of service”)**

**-----------------------------------------------------------------------------------*6. Incorrect tense in abstract ("this study solely surveyed" → "examined"). Author "Younghun B" in [9] should be "B, Y."; DOI in [13] contains extra spaces.***
Response 6：

Thank you for your wise comment. I'm glad to hear that you've made those corrections. It's important for your work to be polished and clear, especially in an abstract where first impressions matter. Therefore, I have corrected the tense problem in the abstract as suggested. Additionally, I've changed the author's name to an abbreviation in the references section and removed any extra spaces in the DOI entries. If you need further assistance or have any more questions about formatting references or anything else, feel free to ask!

**The revised content is as follows (page 2; page 22):**

**-----------------------------------------------------------------------------------**

**-** this study **examined** the ideal travel time of a passenger or cyclist in the hybrid-congested roads (congested and non-congested road accounts for half).

**-** Besides, the various road section’s capacity was **examined**.

**（See line 8-9, 11 in “Abstract”)**

**-** This **experiment attains** 9 **effective hyper-paths with traffic flow and travel impedance attributes attached to each hyper-path**. The scatter chart results show that the **plural modulus** of carrying capacity **decreases** with travel time**’s descends**; when the road saturation **increases** from **0.7** to 0.9, **the plural modulus** **of** carrying capacity **declines** by **27.1%**. Meanwhile, **raising** the **upper limit of expansion** would slightly lift the carrying capacity **in the proportion of 15.7%**. This **research** could provide a reference for the **majorization** of multi-modal cyber flow distribution and also has indirect significance for route planning to a certain extent.

**（See line 15-21 in “Abstract”)**

9.	**B, Y** and Michael H. Household activity pattern problem with automated vehicle-enabled intermodal trips. Transportation Research Part C, 2025, 170:104930. https://doi.org/10.1016/j.trc.2024.104930

13.	Torbjorn Larsson and Michael Patriksson. An augmented lagrangean dual algorithm for link capacity side constrained traffic assignment problems. Transportation Research Part B: Methodological, 1995, 29(6):433–455. https://doi.org/10.1016/0191-2615(95)00016-7

**（See line 458-459, line 473-475 in “References”)**

**-----------------------------------------------------------------------------------**
