# Supplementary material for: Modelling and improving approach for carrying capacity in multi-modal super network by considering travel time
Source: PLoS One. 2025 Dec 22;20(12):e0339039. doi: 10.1371/journal.pone.0339039 (PMC12721534; doi:10.1371/journal.pone.0339039)
Supplement: Attachment — Submitted filename: Responses-reviewer #2.docx [file pone.0339039.s005.docx]

**PLOS One**

**Ref. No.: PONE-D-25-28716**

**Title: Modelling and Improving Approach for Carrying Capacity in Multi-modal Super Network by Considering Travel Time**

Xiangyue Huang<https://orcid.org/0000-0002-9016-6105>^*^

College of Transportation Engineering, Chang'an University, Xi'an,

Shaanxi Province, China

Email: [hxyue019@163.com](mailto:hxyue019@163.com)

**REPLY to reviewer’s comments**

**To Reviewers:**

*Dear Reviewers:*

*The author has thoughtfully addressed each of the reviewers' suggestions and expresses sincere gratitude to both reviewers for their invaluable feedback on the 2^nd^ manuscript. The depth and rigor of the reviewers' analyses, as evidenced by the questions raised, highlight their significant practical relevance. After careful consideration, the responses to these points have been incorporated into the revised version based on the previous draft. The response letter accompanying the revision will include solutions directly within each textual explanation, accompanied by pertinent references. The 3^rd^ revised manuscript will also continue to rectify any inappropriate phrasing identified in the article. Feedback from both reviewers regarding textual revisions is summarized as follows: The revised paper employs the present tense, past tense, and past perfect tense for narrative clarity. Primary, secondary, and tertiary headings are formatted in Times New Roman 18pt, 14pt, and 11pt, respectively, while figure and table captions consistently utilize Times New Roman 10pt.*

*Sincerely,*

*Xiangyue Huang*

**To Reviewer #1 #2**

**Reviewer #1 #2:**

1. ***The title of the article does not highlight the original intention and connotation of modeling in reflecting the research methods of the paper. Therefore, the revised title of the latest revised article is as follows (page 1):***
   Response 1：

Modelling and Improving Approach for Carrying Capacity in Multi-modal Super Network by Considering Travel Time.
**（See line 1-2)**

**To Reviewer #2**

**Reviewer #2:**
***1. Does this conclusion imply that traffic flow should be actively restricted during peak hours to maintain lower saturation?***
Response 1：

Indeed, once the validity of the conclusion has been verified, network capacity should be enhanced by proactively restricting traffic flow during peak periods, while simultaneously redistributing traffic across road sections during off-peak hours to alleviate congestion. The traffic restriction mentioned in the question refers to a passenger transport management measure implemented on urban roads where severe vehicle congestion occurs at junctions. Its purpose is to ensure pedestrian crossing safety and separate motorized and non-motorized traffic. Its principle involves controlling vehicle speeds to reduce the number of vehicles entering lanes per unit time. This measure retains universal applicability in major cities despite increasing traffic volumes, and is being suitable for diverse scenarios including motorways, toll gates, and suburban roads. Various theories exist regarding the origin of flow restriction measures: the traffic system resembles a pipeline whose capacity remains fixed during specific time periods. Congestion occurs when traffic demand surges sharply within a short period and exceeds capacity. During morning and evening rush hours, or when motorway tolls are waived on public holidays, traffic volume forms a sharp ‘peak’, as illustrated in Figure 1. This peak far exceeds the road's normal carrying capacity. Off-peak traffic restrictions, through enforcement or guidance, flatten this sharp ‘peak’ into a more gradual ‘plateau’. While travel speeds may not reach optimal levels, this approach prevents complete system collapse and the vicious cycle of stop-start congestion. The objective is not to enable the fastest passage for every individual, but to ensure the entire system remains operational, maximizing overall throughput while minimizing time costs. From a long-term perspective, it can also optimize the city's overall travel structure: peak-shifting traffic management, particularly stringent vehicle restriction policies, inevitably increases the cost and uncertainty of private car travel. This effectively ‘compels’ a portion of the population to shift towards more intensive travel modes such as public transport, carpooling, and non-motorised vehicles.

Fig 1. Flow distribution during commuting travel times

As outlined in Question 1, while the principles of off-peak and peak-hour traffic restrictions remain broadly consistent across different modes of transport, subtle differences persist in their implementation. The core strategy hinges on two key objectives: firstly, employing economic incentives, administrative directives, and technical measures to reduce the number of motor vehicles—such as buses and taxis - in specific areas during designated times; secondly, enhancing overall traffic efficiency, alleviating congestion, and reducing exhaust emissions by restricting usage times, zones, or eligibility criteria. In contrast, regulations for shared bicycles focus on standardizing riding behaviour—such as ensuring safe travel spaces and methods to enhance the cycling experience - with the primary emphasis on protecting ‘people’. Specific measures include creating physical infrastructure and rule-based guidance to establish safe, continuous, and dedicated travel lanes for shared bicycles as described in the text. This approach differs from segmented pricing strategies to restrict flow, yielding slightly weaker results than directly installing barriers separating motorized and non-motorized traffic. While both off-peak and peak-hour flow restrictions aim to manage traffic volume, their core philosophies, implementation methods, and ultimate outcomes differ fundamentally. The former prioritizes ‘guidance,’ functioning like an intelligent navigation system; the latter focuses on ‘blocking,’ resembling a simple valve. When combined, these approaches yield immediate results in managing traffic flow within major cities.

Numerous traffic restriction measures abroad predominantly adopt the approach of imposing higher ‘tolls’ on passing motorists. For instance, Singapore employs an Electronic Road Pricing (ERP) system featuring congestion pricing, installing ERP gates in central urban areas and on motorways. When vehicles pass through during peak congestion periods, fees are automatically deducted from their prepaid cards. Rates dynamically adjust based on time and congestion severity, peaking during rush hours. This directly charges drivers for ‘creating congestion,’ effectively moderating peak-period traffic flows. London's policy authorities require vehicles entering specific central zones to prepay a fixed daily charge, significantly reducing city centre traffic volumes and boosting bus speeds. The Stockholm government adopted a more ‘humane’ approach—implementing a seven-month congestion tax trial as early as 2006. Results showed over 20% reduction in city centre traffic flow and markedly improved air quality. Building on this successful trial data, Stockholm residents subsequently voted in a referendum to permanently adopt the policy. The strategy of ‘high pricing during peak congestion’ is not applicable to Chinese citizens. On the contrary, holiday travelers prioritize ‘swift and economical’ return journeys over ‘safe’ travel. Consequently, manually operated toll lanes offering free passage nationwide better suit their needs. When entering motorways toll-free and subsequently selecting ETC lanes upon exit, drivers in China may encounter the predicament of lacking recorded information, with exit barriers failing to lift, ultimately forcing them to return to manual lanes for clearance. In contrast, the United States, with its extensive implementation of HOT lanes, has supplemented infrastructure to permit single-occupant vehicles to pay for using express lanes traditionally reserved for multi-occupant vehicles. This facilitates market-driven allocation of road resources, catering to drivers willing to pay for time savings. New York's conversion of Broadway through Times Square into a pedestrian plaza, completely banning motorized traffic, demonstrates how bicycle rights can be secured in densely built-up urban environments. Overall, bicycle flow management prioritizes demand control. Technologically, domestic approaches typically outperform foreign public-private partnership schemes. This is because overseas models, once caught in fiscal crises where customers struggle to pay annual rental fees, often see developers' revenue targets fall short of projections. China's advantage in bicycle demand management lies in more precise GPS latitude/longitude positioning and stricter penalty mechanisms for incorrect parking. It avoids the phenomenon where ‘after a vehicle is returned and locked, its real-time location cannot be transmitted back to the system, causing the system to frequently “lose track” of vehicles.’ Consequently, vehicle supply locations and distribution remain balanced. Even on secondary roads and side streets in urban-rural fringe areas, traffic restriction measures prove highly effective.

Now, regarding the argument raised: ‘Does this conclusion imply that traffic flow should be proactively restricted during peak hours to maintain lower saturation levels?’ Firstly, this conclusion is a scientifically validated logical inference. The original text states: ‘When saturation increases by 0.1, the scatter plot shifts further from the origin, indicating a declining trend in carrying capacity.’ Multiple refinements to the genetic algorithm's operations demonstrate that— under three service level conditions—the Pareto frontier's fitted curve and its 95% confidence interval band are clearly discernible. Secondly, taking bridges and tunnel sections within Chongqing's central urban area as an example, these locations are typically classified as accident-prone zones due to narrow lanes and limited evasion space. Implementing staggered traffic flow strategies helps maintain lower saturation levels of traffic flow within the network, thereby ensuring stable and orderly traffic operations in the central urban area.

***2. Is the cost-benefit analysis of widening key road sections (such as the 10 sections mentioned in the article) sufficient to support their prioritization as improvement projects? Have other intervention measures (such as signal optimization and bus priority) been considered?***
Response 2：

I consider the viewpoint in Question 2 to be overly generalized. While a cost-benefit analysis of the expansion demonstrates the project's compliance with specifications and serves as an analytical case study for the construction cost industry, it does not prove that the expansion will provide timely relief for the urgent issue of network capacity constraints, nor does it necessarily constitute the preferred improvement solution. The text describes the improvement scheme aimed at minimizing capital expenditure as follows: ‘the change in carrying capacity has reached 2276.8 pcu/h. Ultimately, the expansion cost consumed was fixed at 13505.74, with a unit of ten thousand yuan.’ This implies that widening critical road sections entails excessive expenditure, often unchecked. In practice, multiple factors must be considered: the number of road structures (bridges, tunnels, etc.) along the carriageway direction; spatial areas with high pedestrian volumes; and real-time monitoring and analysis of average vehicle speeds and traffic density. This constitutes a substantial undertaking, demanding considerable time and manpower. In summary, when determining improvement plans, the initial step should involve collecting and collating survey data for each traffic district, alongside formulating investigation plans for each district and county. Based on these plans, traffic management authorities must articulate the project's background, significance, and feasibility, analyzing its viability from technical, economic, and policy perspectives. This ensures compliance with approval documents and standards. Subsequently, determine the layout of new roads, classify them into arterial, secondary arterial, and collector roads, and conduct project cost estimates based on the modification proposals. Finally, after multi-level departmental verification, the project is approved. Upon entering the implementation phase, the contractor supervises the orderly progress of the project, manages and controls the construction schedule, and ensures its timely completion in accordance with the design requirements and quality standards specified in the construction drawings.

The paper proposes 21 model parameters and 20 model variables entirely. The cost-benefit analysis for widening key road sections primarily encompasses two components: earthworks development and integrated combination schemes. Consequently, the total expansion cost is calculated cumulatively using a fixed unit pricing method per lane widened. The effectiveness of combined solutions with varying expansion widths is compared, naturally yielding the conclusion that ‘greater widening depth results in higher construction costs,’ thereby achieving optimal resource allocation within finite constraints. To further leverage the unique hierarchical ‘leader-follower’ structure of the dual-layer planning model and highlight the power differential between objectives and constraints, a sensitivity analysis was conducted on the upper-layer optimization model. This involved altering the upper widening threshold *k_b_* for individual lanes to compare expansion investment levels, with sensitivity tests conducted in increments of 50 pcu/h. Using *k_b_*=200 pcu/h as the control group, the comparative findings offer an alternative approach for prioritizing improvement projects. Although the solution process is relatively cumbersome, the complexity of this modelling approach is often justified to obtain genuinely feasible and effective decision-making solutions in the real world. It is thus evident that the role of cost-benefit analysis in engineering improvement projects is to allocate resources towards initiatives yielding the greatest net social benefit or highest return on investment. This ensures resources are deployed where they are most effective, preventing waste on inefficient or ineffective projects. From a long-term development perspective, the benefits derived from enhancing carrying capacity do not stem from direct financial income but rather from intangible, long-term gains. The pathways to such enhancement play a pivotal role in managerial decision-making. These intangible outputs encompass social benefits, environmental benefits, and more, collectively exerting the most sustainable and value-creating positive influence in expanding the scope for carrying capacity improvement. Having conducted an in-depth analysis from an engineering materials perspective, major cities with low-lying geological topography tend to utilize cement concrete for road surfacing, costing approximately ¥30 million per kilometre. In contrast, gravel-based road surfacing incurs costs reaching around ¥80 million per kilometre. Although asphalt surfaces carry a higher initial cost than concrete, their superior durability and resilience, coupled with simpler long-term maintenance, render them more cost-effective over time. Factoring in the production and transportation costs of limestone, the overall expansion plan could incur expenditures in the tens of millions. Traffic light installations - comprising signal units, control lines, and lampposts. Including transportation, installation, commissioning costs, and taxes, the total investment represents merely 1% to 5% of the expansion plan. Therefore, engineering improvement schemes based on flexible rather than rigid measures yield more economical cost-benefit outcomes. This somewhat contradicts the conclusion in Question 2, namely that ‘for key sections, such as the ten critical widening roads mentioned in the text, conducting a cost-benefit analysis does not sufficiently support their justification as priority improvement projects.’

In the revised paper, congestion on certain lanes is treated as a constraint condition for the upper-layer model within the two-layer framework. This is integrated with the lower-layer model's user flow equilibrium allocation algorithm to monitor the state of network traffic flows. Beyond conventional congestion mitigation strategies, network optimization interventions based on capacity expansion may be analyzed from four perspectives: firstly, refined signal control to unlock existing potential; secondly, intensified public transport to enhance transport efficiency; thirdly, strategic infrastructure expansion to develop multi-modal transport systems; and fourthly, integrated mobility services to advance intelligent technologies.

The approach adopted in this paper represents the third perspective for enhancement. The first perspective encompasses the installation of sensor-activated traffic lights for non-motorized lanes, the proposal of a flow-adaptive signal coordination system, and signal optimization for ‘left-turn double crossing’ manoeuvres. The detectors within the sensor-based signal system resemble the camera-based enforcement equipment at checkpoints. Their core component—the microwave radar vehicle detector (Figure 3)—demands exceptionally high speed measurement accuracy, with national standards requiring an error margin within -6%. At speed cameras, two photographs of the vehicle at different positions are captured during speed measurement. The speeding percentage is calculated based on the intersection's speed limit and the vehicle's actual speed, with penalties imposed according to regulations. For wrong-way driving detection, capturing the entire violation process is equally essential. At one-way intersections, the yield sign must be clearly visible in the captured image. Beyond conventional number plate recognition, the camera-based system identifies vehicle size, body type, and manufacturer badges. It must also provide clear 24-hour visibility of the driver's facial features. When detecting non-motorised vehicles awaiting left turns or straight passage, the sensor-controlled traffic system automatically extends the green light duration or adds a dedicated phase, as illustrated in Figure 2.

Fig 2. Intelligent intersection based on induction signal light control

It is worth noting that most traditional capacity enhancement strategies focus on network-level distribution. For instance, concerning delays at roads and junctions, the formulation of signal control optimization strategies linking arterial roads with multiple intersections increases the number of vehicles smoothly passing through junctions while reducing the probability of single-lane congestion. Based on the aforementioned precise sensing data, regional signal control systems can detect non-motorized traffic flow ‘tide’ phenomena in real time, such as morning and evening rush hour tides. Dynamically adjusting signal timing schemes across a series of junctions can create ‘green waves’ or facilitate rapid traffic dispersal. Signal optimization for ‘two-stage left turns’ addresses the hazards of non-motorized vehicles making left turns at large intersections. Through precise traffic light control, this ensures the safety and efficiency of two-stage crossing manoeuvres.

The second perspective encompasses strategic approaches such as “public transport priority”, “TOD development”, and “public transport network planning”. The “public transport priority” strategy positions public transport at the core of the integrated urban transport system, ensuring priority in urban planning, road rights allocation, and funding allocation. The “TOD model” refers to transit-oriented urban development. Policy guidance directs high-density, mixed-use urban development around bus stops, or metro and BRT stations, thereby generating passenger flows at source. ‘Public transport network planning’ establishes a scientifically sound network comprising trunk, feeder and micro-loop routes, avoiding duplication or gaps in coverage. Its contribution lies in enabling a single bus or metro carriage to replace dozens or even hundreds of private vehicles. Effective management policies, such as dedicated bus lanes, not only prioritize higher-capacity transport modes within existing road space but also incentivize more pedestrians to choose public transport. This fundamentally reduces the total number of motor vehicles on roads, directly alleviating pressure on the entire transport network. This approach represents not merely “expansion” through additional road construction, but a fundamental “efficiency gain” achieved by enhancing passenger transport capacity per unit of road resource.。

The fourth analytical perspective—“Expanding Intelligent Technologies”—reminds us that we have entered the complex era of Artificial Intelligence (AI) and the Internet of Things (IoT). The emergence of these emerging technologies will transform the lives of human drivers, playing an indispensable role in managing and streamlining traffic networks. The integration of radar systems with acoustic and visual technologies enables precise vehicle positioning. In essence, when toll station radars detect potential collision risks, ground-level LED projections automatically display warning patterns or emit audible alerts to caution both parties. This achieves safe traffic diversion and behavioural guidance within constrained physical spaces through technological means.

Fig 3. 15—Millimeter wave radar control unit

The data acquisition process involves mounting the radar module at the rear of the vehicle, such as on the rear bumper or mid-section of the tailgate, ensuring the radar can effectively gather rear vehicle speed and distance information. This acquired data is then transmitted via the Controller Area Network (CAN) to the vehicle's central control unit. In future, augmented reality (AR) navigation will provide cyclists with virtual guide lines superimposed upon the real road surface via mobile applications or smart helmets, directing them towards optimal and safest routes. The “bus priority” strategy achieves a shift from “incremental expansion”—merely adding vehicles—to “quality enhancement”—improving system operational efficiency—by allocating limited road resources towards more efficient public transport. Without priority measures, buses share roads with private vehicles, falling into a ‘congestion trap’: they wait at red lights and endure traffic jams like private cars, resulting in low travel speeds. Congestion also causes unpredictable arrival times and erratic service intervals, leading to slow operation and poor punctuality, which in turn reduces the passenger turnover efficiency per bus trip. Bus priority strategies play a fundamental and decisive role in enhancing the capacity of public transport systems. Their impact extends beyond merely deploying additional vehicles; through systematic optimization, they fundamentally improve the efficiency, reliability, and attractiveness of bus networks, thereby achieving a significant increase in overall capacity.

For future-oriented advanced technologies, enabling low-cost communication between vehicles, infrastructure and road users is paramount. V2X (Vehicle-to-Everything) technology endows vehicles with foresight and predictive capabilities. Through proactive, seamless decision-making, it prevents sudden braking and traffic flow disruption caused by unforeseen incidents, thereby maintaining overall road capacity. When most vehicles possess V2X capabilities (Figure 4) and highly automated driving functions, traffic management systems can act like an ‘air traffic controller,’ globally optimizing and coordinating regional traffic flows to enhance the capacity of the transport system. In my view, V2X/ITS (Intelligent Transportation Systems) technology functions more like a commander, intelligently allocating travel speeds and braking distances across different vehicle types. For instance, when the vehicle ahead initiates braking, the following vehicle receives the signal almost instantaneously and begins its response, significantly reducing driver reaction time. Moreover, it enables vehicles to maintain shorter yet safer following distances at high speeds, thereby accommodating more vehicles within a single lane. This indirectly enhances the carrying capacity of the entire transport network.

Fig 4. Signal transmission based on V2X

3. ***Author’s dual publication concerns, research ethics, or publication ethics statement.***

Responses 3:

Firstly, the author of this paper is the primary drafter of the original manuscript and has made significant contributions to the research methodology, preparation of figures and tables, acquisition of required data, and analysis of results. The published version is hereby finalized at this stage. The author agrees to assume responsibility for all aspects of the research work to ensure that any queries regarding the accuracy or integrity of any part of the paper are properly investigated and resolved. I, along with postgraduate students and teaching assistants within my research group, resolutely oppose academic misconduct: Firstly, plagiarism and copying; scholars must not utilize others' work without the original author's consent or without proper citation or acknowledgement. Secondly, falsification and fabrication; under no circumstances shall data, conclusions, or other elements be fabricated or falsified, including those derived from non-experimental or non-research sources. Thirdly, fragmentary publication, duplicate publication, and patchwork publication; fragmenting the same research for separate publication; submitting the same manuscript to multiple journals or publishing it repeatedly; or compiling content from multiple previously published independent works into a single publication. Out of trust in journal publishers, these serious violations of research ethics have been blacklisted by myself and are strictly prohibited by all members of my team.

Secondly, this study aims to evaluate the impact of user travel time on enhancing network capacity. The research context stems from the complex multi-modal vehicle flow patterns within major urban networks, necessitating a clear understanding of travel characteristics across all transport modes. The primary objective is to validate and evaluate the effectiveness and sensitivity of the proposed two-layer model. This involves comparative trials on key road widening projects, where initial network demand will be evenly distributed between experimental and control groups. The experimental group will receive engineering improvements, while the control group will maintain conventional traffic distribution operations. Throughout the study, all traffic flows will adhere to the statistical model of discrete choice analysis.

Furthermore, this research has gained recognition from several leading scholars in the field of transport engineering. The data employed in this study comprises field survey statistics, computational outputs from an enhanced genetic algorithm, and data gaps derived from the evolution of computational conclusions. The acquired data encompassed traffic network equilibrium distribution data derived from continuous iterative algorithms, alongside bearing capacity modulus results computed via the NSGA-II (Non-dominated Sorting Genetic Algorithm II) methodology. Moreover, the extracted data underwent validation and verification by Dr Ziyou Gao, Dr Yan Li, and Dr Bingfeng Si to ensure data accuracy. The database is publicly accessible with a waiver for ethical approval statements and informed consent. Key risks in this study include travel delays and infrastructure damage during extreme weather events; however, all experiments were conducted under normal weather conditions and ideal environmental settings, with computational results obtained through programming specifications. Potential benefits include providing more effective economic project proposals for network expansion.

Finally, certain sections of the article potentially connect with personal privacy, data protection, and subject rights shall undergo ethical review by the Ethics Committee to assess the moral justification of the research project. This safeguards the rights and welfare of research subjects, prevents potential physical, psychological, or social harm, and ensures the research process remains transparent, compliant, and reasonable. It should be clarified that no conflicts of interest exist among the researchers involved in this study.

**References：**

[1]	Wang Z ,Zheng X ,Meng F , et al. Exploring the Joint Influence of Built Environment Factors on Urban Rail Transit Peak-Hour Ridership Using DeepSeek [J]. Buildings, 2025, 15 (10): 1744-1744.

[2]	Li J ,Zhang Y ,Yu S , et al. AI-Driven urban planning optimization: A graph neural network and genetic algorithm framework for tackling peak-hour challenges [J]. Sustainable Cities and Society, 2025, 126 106407-106407.

[3]	Liu L ,Liu Y ,Ye X . Multi-sequence spatio-temporal feature fusion network for peak-hour passenger flow prediction in urban rail transit [J]. Transportation Letters, 2025, 17 (1): 86-102.

[4]	Li Z ,Zhao H ,Zhao J , et al. SAT-Net: A staggered attention network using graph neural networks for encrypted traffic classification [J]. Journal of Network and Computer Applications, 2025, 233 104069-104069.

[5]	Chuanyao L ,Yichao L ,Gege J , et al. Social optimum for linear staggered shifts in a single-entry traffic corridor with no late arrivals [J]. Transportmetrica B: Transport Dynamics, 2023, 11 (1): 630-648. DOI:10.1080/21680566.2022.2113176.

[6]	Astana Y N I ,Sudarsana K D ,Frederika A , et al. Development of contingency cost analysis model in construction project with cost-time-quality risk integration [J]. Eastern-European Journal of Enterprise Technologies, 2025, 3 (3 (135)): 49-56.

[7]	Kweek A L ,Arumsari P . Acceleration Alternatives Cost Analysis of Train Station Construction Project [J]. IOP Conference Series: Earth and Environmental Science, 2025, 1488 (1): 012045-012045.

[8]	Altaf M ,Jaffari R ,Alalaoul S W , et al. Developing automated strategy of life cycle cost analysis (LCCA) with building information modeling (BIM) integration for building projects [J]. Results in Engineering, 2025, 25 104179-104179.

[9]	Muhammad A ,Salah W A ,Ali M M , et al. Life cycle cost analysis (LCCA) of construction projects: sustainability perspective [J]. Environment, Development and Sustainability, 2022, 25 (11): 12071-12118. DOI:10.1007/S10668-022-02579-X.

[10]	Yuhong G ,Zhaowei Q ,Xianmin S , et al. Coordinated perimeter control of urban road network based on traffic carrying capacity model [J]. Simulation Modelling Practice and Theory, 2023, 123.
